# The Efficacy of Nebulized Furosemide and Salbutamol Compared with Salbutamol Alone in Reactive Airway Disease: A Double Blind Randomized, Clinical Trial

**DOI:** 10.1155/2014/638102

**Published:** 2014-04-27

**Authors:** Kambiz Masoumi, Arash Forouzan, Maryam Haddadzadeh Shoushtari, Samaneh Porozan, Maryam Feli, Mehdi Fallah Bagher Sheidaee, Ali Asgari Darian

**Affiliations:** ^1^Department of Emergency Medicine, Imam Khomeini Hospital, Ahvaz Jundishapur University of Medical Sciences, Ahvaz 6193673166, Iran; ^2^Department of Internal Medicine, Division of Pulmonology, Imam Khomeini Hospital, Ahvaz Jundishapur University of Medical Sciences, Ahvaz 6193673166, Iran

## Abstract

We undertook this randomized clinical trial to investigate whether adding furosemide to salbutamol could improve the peak expiratory flow rate (PEFR) and clinical signs of reactive airway disease (RAD) patients. Eligible 18- to 55-year-old patients were randomly divided into intervention and control groups. Patients received 5 mg of nebulized salbutamol and 40 mg of nebulized furosemide in the intervention group and 5 mg of nebulized salbutamol alone in the control group. Patients in both groups received 100 mg of methylprednisolone intravenously stat. Severity of the RAD was estimated before and 45 minutes after treatment in both groups. PEFR was estimated before treatment and at 15, 30, and 45 minutes later. Ninety patients were enrolled, 45 in each group. There were no significant differences between two groups regarding gender, mean age, and normalized PEFR. The baseline mean PEFR was not significantly different between groups (*P* = 0.58). A repeated measure analysis of variance revealed that the differences between the two treatments was significant (*P* = 0.0001) and the behavior of two treatments was not similar across the time (*P* = 0.001). Comparison of clinical severity of acute RAD revealed no significant differences between groups at the end of the trial (0.06). This study showed that adding nebulized furosemide to salbutamol in RAD patients improved PEFR.

## 1. Introduction

A lot of patients complaining of dyspnea present to the emergency department. Dyspnea is a common symptom of many illnesses, which is defined as an unpleasant respiratory sense [1]. It can adversely affect the quality of life. A widespread cause of dyspnea is respiratory system dysfunctions such as asthma, COPD, pneumonia, and bronchitis [2, 3]. Besides, a number of patients presenting with dyspnea do not have an established diagnosis. These patients are considered as reactive airway disease (RAD) patients. These cases have a history of cough, sputum production, wheeze, dyspnea, or history of inhaler use. Most often, physicians who use the term “reactive airways disease” do not have the results of pulmonary function test for these patients. In the pediatric setting, especially in very young children, the term “reactive airways disease” may be used as a nonspecific term in clinical contexts ranging from asthma to wheezy bronchitis, to viral bronchiolitis, or even to pneumonia. In adult medicine, we suspect that the term is popular because of instances in which physicians obtain a history of wheeze, sputum production, or inhaler use, but a formal diagnosis of asthma is not in the patient record. Frequently, the physiological information is missing or elements of a typical asthma history are missing. In the absence of these findings, physicians will provide a label of “reactive airways disease” to convey that the patient has some sort of airway problem. These patients may actually have asthma, chronic bronchitis, emphysema, or even pneumonia [4].

Although some publications have used the term RAD to describe patients with asthma and/or COPD or synonymously with airway hyperreactivity [5, 6], it seems that this trend is troubling because many patients considered to have RAD do not have asthma, and the vast majority of patients with reactive airways have never had their airway reactivity measured. Medication usually prescribed for these specific diseases may or may not be prescribed if the diagnosis is RAD. It is suspected that many patients with a diagnosis of RAD receive inhaled *β*-agonists or inhaled corticosteroids [7, 8]. The term RAD needs to be distinguished from reactive airways dysfunction syndrome (asthma-like illness developing after a single exposure to high levels of an irritating vapor, fume, or smoke) [9].

Apart from the underlying etiology of dyspnea, an effective and safe treatment strategy should be considered. Nebulization, as a quick acting and easy method of drug administration, is becoming more popular. There are many drugs that are simply taken nebulized [10]. The drugs must reach the intended location and remain active to obtain satisfactory outcomes [11].

Many studies have been conducted on the effect of using nebulized furosemide alone or along with other standard treatments for patients who often suffer from dyspnea for different reasons. These studies have suggested that nebulized furosemide, a loop diuretic, can exert a bronchodilatory effect [12] and has been used in combination with beta-agonists in the treatment of bronchial asthma and chronic obstructive pulmonary disease [13, 14]. Moreover, it could be effective in relieving dyspnea in cancer patients [15] and decreasing experimentally induced dyspnea in healthy subjects [16].

Although the potential benefits of nebulized furosemide have been reported, the clinical evidence to support its addition to standard therapy is insufficient [17]. According to the conflicting results of previous studies, we performed a randomized clinical trial to investigate whether adding furosemide to salbutamol or not could improve the peak expiratory flow rate.

## 2. Materials and Methods

Patients who were 18–55 years old with dyspnea, cough, and wheezing or history of using the inhaler, attended to the emergency department of Imam Khomeini Hospital, Ahvaz, Iran, from October 1, 2012, to April 1, 2013, and were labeled with an acute attack of possible reactive airway disease were included. Imam Khomeini Hospital in Ahvaz is a 500-bed teaching and referral hospital with specialty and subspecialty departments in many fields of clinical medicine. Almost 100,000 patients are visited in its emergency department per year. Ahvaz is the largest city and the capital of Khuzestan province in southwest of Iran.

This study was a double blind study of parallel groups of patients with RAD. Recorded formal diagnosis of asthma or COPD, noncardiac pulmonary edema, symptoms related to inhalation of irritant gas, aerosol or smoke, long duration of symptom onset (>10 hour), smoking more than 10 packs/year, comorbid acute medical problems, pregnancy, and administration of nebulized beta-agonist in the previous 6 hours were considered as exclusion criteria.

Eligible patients were randomly divided into intervention and control groups using the block randomization method. In both groups, the severity of the RAD (PEFR_*p*_ < 40% PEFR_*Max*⁡_ = severe, 40% PEFR_*Max*⁡_ < PEFR_*p*_ < 70%  PEFR_*Max*⁡_ = moderate, and PEFR_*p*_ > 70%  PEFR_*Max*⁡_ = mild) (PEFR_MAX_ = maximum predicted PEFR based on gender, age, and height in normal situation, PEFR_40%_ = 40% of maximum predicted PEFR, PEFR_70%_ = 70% of maximum predicted PEFR, and PEFR_*P*_ = mean measured PEFR of patients by peak flow meter in emergency department) and peak expiratory flow rate (PEFR) were estimated and recorded based on the history, physical examination, and peak flow metry before treatment. Then, in the intervention group, 5 mg of salbutamol (Cipla Ltd. India/Kimiara Heram, Tehran, Iran, 2.5 mg/2 cc) and 40 mg of furosemide vial (Chemidarou Industrial Company, Tehran, Iran, 20 mg/2 cc) were nebulized for the patients during 15 minutes and, in the control group, 5 mg of salbutamol alone was nebulized for the patients during 15 minutes. The PEFR was measured in every patient before nebulization and in the 15, 30, and 45 minutes after it. The severity of the RAD was estimated 45 minutes after nebulization again. Patients of both groups received 100 mg of methylprednisolone (500 mg/vial as sodium succinate) intravenously stat. If any patient did not respond to the treatment and their general condition was aggravated, other lines of treatment (MgSO_4_ IV, epinephrine IM,…) were tried or the treatment was repeated. Such subjects were excluded from the trial. For nebulization, an ultrasonic nebulizer was used (*ɱ* SUCHATZKI Germany/Medika, Tehran, Iran; Micro 800 XX series). For peak flow metry, a digital peak flow meter was used after calibration (Cegla GmbH & Co. KG. Germany/NabzHayat, Tehran, Iran; HRC-test asthma). At each recording of PEFR, the patients were asked to perform peak flow meter three times. Then, the highest level was recorded for each patient. Based on height, age, and gender, PEFR_*Max*⁡_ and subsequently PEFR_70%_ and PEFR_40%_ were calculated and the clinical severity of the disease (severe, moderate, and mild) was estimated based on measured PEFR in comparison with predicted PEFR as mentioned above.

A written informed consent was obtained from all subjects. This study was confirmed by the Ethics Committee of Ahvaz Jundishapur University of Medical Sciences. Besides, this work was conducted in accordance with the Declaration of Helsinki 1964.

### 2.1. Statistical Analysis

Data were summarized as mean ± SD. The repeated measures test, independent *t*-test, and Chi-squared test were used for data analysis. All reported *P* values less than 0.05 were considered statistically significant.

## 3. Results

Ninety patients met the inclusion criteria and were enrolled in the study. During the study period, none of patients were excluded from the study because of unresponsiveness or deterioration. They were allocated in a random fashion, using block randomization, into the intervention group (salbutamol and furosemide) and the control group (salbutamol alone), with 45 patients in either group. No significant differences were identified between the subjects of the two groups about basic demographic data including age and gender, PEFR_*Max*⁡_, PEFR_40%_, and PEFR_70%_ (*P* values <0.05) (see Table 1). Also, mean PEFR of patients in two groups was not significant before treatment in zero minutes (salbutamol group: 234.42 ± 67.487 and  salbutamol and furosemide group: 243.38 ± 87.608 (*P* value =0.58)).

**Figure 1:**
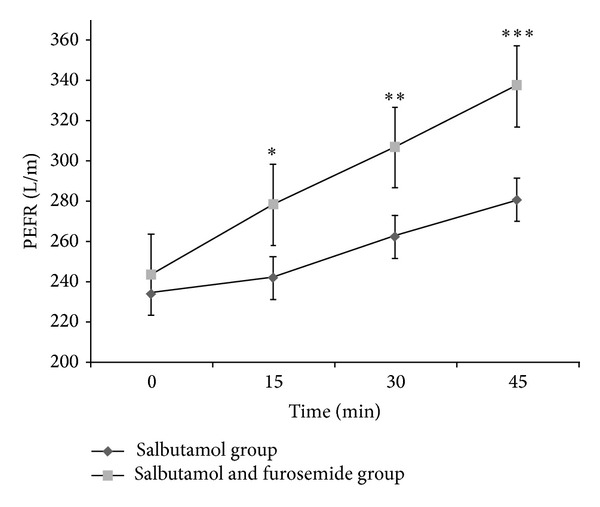
Comparison of mean (±SD) peak of the expiratory flow rate (PEFR) of nebulized salbutamol (*n* = 45) and nebulized salbutamol with furosemide (*n* = 45) based on time. (*P = 0.03, **P = 0.01, and ***P = 0.001).

The mean ± SD PEFR at minutes 0, 15, 30, and 45 of the two groups of participants is shown in Figure 1. A repeated measure analysis of variance revealed that the differences between the two treatments were significant and PEFR improvement in all end points from 15 min to 45 min after intervention was significantly higher in the furosemide group as shown in Figure 1.

**Figure 2:**
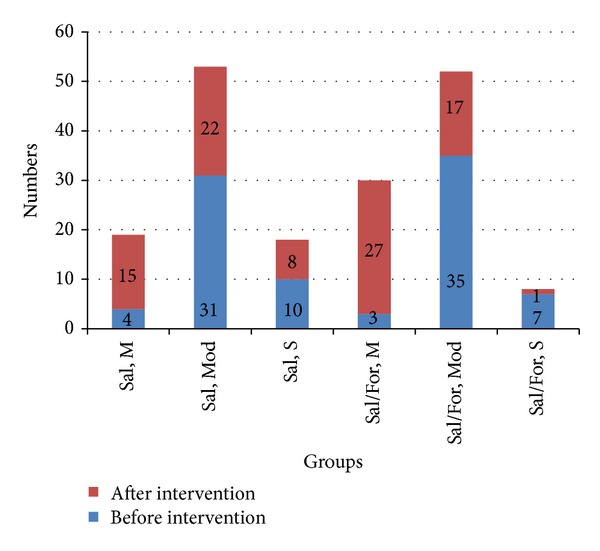
Bar chart of the number of patients in each group with mild, moderate, or severe dyspnea and comparison of these numbers before and after intervention. (Sal, M: salbutamol, mild, Sal, Mod: salbutamol, moderate, Sal, S: salbutamol, severe, Sal/For, M: salbutamol/furosemide, mild, Sal/For, Mod: salbutamol/furosemide, moderate, and Sal/For, S: salbutamol/furosemide, severe).

The difference between the mean PEFR of the two groups was significant at the end of the trial (280.62 ± 84.384 versus 336.98 ± 81.846, *P* = 0.001). A significant difference was observed in the change of PEFR at 45 minutes compared to baseline in both groups (*P* = 0.001). Most participants in both groups before treatment had moderate dyspnea based on their PEFR. At the end of the trial, the number of patients with severe dyspnea was higher in the salbutamol group (Figure 2).

## 4. Discussion

Treatment of acute reactive airway disease as a debilitating clinical statement is crucial. Conventional treatments of asthma and COPD including *β*-agonists and corticosteroids are considered effective in the RAD symptom improvement. Besides, several studies have assessed the effect of nebulized furosemide in treating dyspnea which is the foremost symptom of RAD [12, 14, 15].

Furosemide increases diuresis due to its simultaneous transmission of sodium, potassium, and chlorine ions in the ascending limb of the Henle loop [18]. The mechanism of inhaled furosemide is still unknown despite the fact that it has been studied extensively. This has fostered the notion that more than one mechanism may be involved including induction of relaxant prostaglandins, blocking mediator production of inflammatory cells, and regulating ionic exchange in the epithelium of the airway [12, 19].

As expected, in this study both groups of patients showed a significant improvement of PEFR 45 minutes after nebulization of intended drug. In agreement with our hypothesis, adding nebulized furosemide to salbutamol in patients suffering from acute RAD with any degree of severity and in the light of the inclusion criteria considerably improved PEFR and clinical signs of the patients. The improvement was statistically significant compared to nebulized salbutamol alone. Meanwhile, no side effect was reported. The results of the present study provide statistically significant support for enhancement of the salbutamol effect by concurrent treatment with furosemide. Clinical characteristics of the patients, such as gender, age, and normalized PEFR, did not differ between the groups and cannot explain differences in the therapeutic outcome.

These findings confirmed the results of a previous study in which Bianco et al. 1988 conducted a trial on the role of oral furosemide in preventing exercise induced asthma compared to nebulized furosemide. They suggested that nebulized furosemide has a direct protective effect on the airway [20]. Besides, the result of the present study is in a line with Chin et al., which reported that the combining of furosemide and albuterol compared with furosemide or albuterol alone has a significant bronchodilatory effect in children with mild asthma [21].

Pendino et al. in a double blind clinical trial in patients with acute asthmatic exacerbation found no significant differences in PEFR between salbutamol/furosemide and salbutamol/saline treated patients 15 and 30 minutes following inhalation. However, when they separately examined patients who had a short duration (<8 hours) exacerbation, PEFR showed significant improvement in salbutamol/furosemide group [19]. Karpel et al. studied the effect of nebulized furosemide in the treatment on 24 patients with acute airway obstruction. They revealed no significant difference in the increase of the forced expiratory flow rate in 1st second (FEV_1_). Nevertheless, our study was conducted on a larger sample size and the disagreement between the two trials could be attributed to this difference. Other studies revealed that adding nebulized furosemide to standard treatment of acute asthma, particularly mild to moderate asthma [18], produced positive results. Other studies verified the role of inhaled furosemide in improving dyspnea in cancer patients [13]. Whereas the therapeutic effects of nebulized furosemide are attractive, it is important to consider the side effects under combination therapy and associated diseases. Some studies reported an increase in diuresis [22, 23] while others found no side effects [12, 24].

In conclusion, the present study showed that adding furosemide to salbutamol in patients suffering from acute RAD considerably improve PEFR but there is not sufficient proof to confirm it as a routine standard treatment of acute asthma or acute RAD or miscellaneous dyspnea. Unfortunately, we did not analyze patients improvement by their age. Thus, we cannot evaluate patients outcome based on their age; this is a limitation of the present study. It is necessary that further studies be conducted to assess the effectiveness, indications, and safety profile of the method.

## Figures and Tables

**Table 1:** Comparison of sex, mean age, mean of PEFR_max_, mean of PEFR_40%_, and mean of PEFR_70%_ between the two groups.

	Salbutamol group	Salbutamol and furosemide group	*P* value
Women	33	27	0.18
Men	12	18	
Age (mean ± SD)	41.38 ± 10.798	37.73 ± 10.116	0.1
PEFR_Max_	467.45 ± 83.64	504.11 ± 98.73	0.06
PEFR_40%_	186.98 ± 33.59	201.64 ± 41.483	0.07
PEFR_70%_	331.05 ± 58.901	355 ± 69.52	0.08
